# Assessing WHO prioritisation criteria for children 6–59 months treated for moderate wasting in a MUAC-based protocol: a multicountry analysis in West and Central Africa

**DOI:** 10.1136/bmjgh-2025-023264

**Published:** 2026-07-10

**Authors:** Maguy Daures, Ibrahim Sana, Wah Coulibaly, Mahaman Nouhou, Susan Shepherd, Renaud Becquet, Kevin Phelan

**Affiliations:** 1 Bordeaux Population Health Research Centre, University of Bordeaux, Bordeaux, France; 2 ALIMA, Dakar, Senegal; 3 ALIMA, Paris, France

**Keywords:** Nutrition, Child health, Africa

## Abstract

**Background:**

In 2023, the WHO recommended prioritising treatment for moderately wasted children with higher risk characteristics, including mid-upper arm circumference (MUAC) 115–119 mm, weight-for-age z-score (WAZ) <–3, age <24 months or clinical and social vulnerabilities. However, applying multiple criteria is operationally challenging. The OptiMA (Optimising Acute Malnutrition Treatment) protocol treats children with MUAC <125 mm or oedema using reduced doses of ready-to-use therapeutic food. This study examined the distribution and programmatic relevance of WHO prioritisation criteria among moderately wasted children in West and central Africa.

**Methods:**

A prospective observational cohort was conducted in 2022–2024 among children aged 6–59 months treated under OptiMA in Ngouri (Chad), Bamako (Mali) and Mirriah (Niger). Children with MUAC 115–124 mm in the higher-priority group (defined by MUAC 115–119 mm, WAZ <–3 or age <24 months) were compared with children in the lower-priority group (none of these criteria) across recovery, non-response, hospitalisation and death. A multinomial logistic regression model assessed associations between individual criteria and treatment failure.

**Results:**

A total of 87 961 moderate wasting treatment episodes were analysed. Overall, 97.4% of children met at least one WHO higher-priority criterion, of whom 42.5% had a MUAC 115–119 mm. Among children with MUAC 120–124 mm, 46.1% were <24 months, 18.7% WAZ<–3 and 30.6% met both criteria. Recovery was high (85%–90%) and mortality was low (<0.4%) in both higher-priority and lower-priority groups. WAZ <–3 emerged as the strongest independent predictor of adverse outcomes.

**Conclusions:**

Nearly all moderately wasted children managed under OptiMA met at least one WHO higher-priority criterion, calling into question the need for further risk stratification in areas using MUAC-based simplified combined protocols. Such protocols may need to be adapted, though, to include other higher-priority children, like those with WAZ <–3 and MUAC ≥125 mm.

**WHAT IS ALREADY KNOWN ON THIS TOPIC**

- The WHO (2023) recommends prioritising treatment for moderate wasting with specialised foods for children presenting with any of eight additional risk factors. Evidence from one simplified protocol (Potani, 2026) suggests that most children with moderate wasting meet at least one of these priority criteria, raising concerns about the feasibility of such prioritisation. Therefore, simplified approaches treating all children with mid-upper arm circumference (MUAC) <125 mm or nutritional oedema in a single programme could be an effective strategy to operationalise WHO guidance but need further exploration.

**WHAT THIS STUDY ADDS**

- This is the first multicountry study to demonstrate that nearly all children (97.4%) admitted for moderate wasting by MUAC (115–124 mm) under a MUAC-based simplified, combined protocol (Optimising Acute Malnutrition Treatment) met at least one of the following WHO higher-priority criteria: MUAC 115–119 mm, age <24 months or weight-for-age z (WAZ) <–3. Despite this higher-priority classification, recovery proportions were high (85%–90%) and mortality was low, suggesting that risk stratification did not predict poorer outcomes in this MUAC-based protocol treating moderately wasted children with ready-to-use therapeutic food. WAZ <−3 emerged as the single strongest independent predictor of adverse outcomes (death, default and non-response).

**HOW THIS STUDY MIGHT AFFECT RESEARCH, PRACTICE OR POLICY**

- These findings support MUAC-based simplified, combined protocols as a pragmatic and effective strategy to operationalise WHO guidance and expand treatment access for children with moderate wasting. Future research should investigate the effectiveness of these protocols for children with WAZ <−3 but MUAC ≥125 mm, a high-risk group excluded from current MUAC-based protocols.

## Introduction

 More than 30 million children 6–59 months of age suffered from moderate wasting worldwide in 2024.^1^ Although not as lethal as severe wasting, moderate wasting is associated with increased risk of morbidity, developmental delays and mortality.^2^ Furthermore, all severely wasted children pass through moderate wasting, with some studies having shown that the percentage of children who deteriorate from moderate to severe wasting in the absence of treatment can range from 10% to 30%.^3^,^5^

The WHO defines moderate wasting as non-edematous children presenting with a weight-for-height z score (WHZ) between −2 and −3 and/or a mid-upper arm circumference (MUAC) between 115 and 124 mm.^6^ In 2023, the WHO provided normative guidance for the first time on the management of children with moderate wasting, including a call to prioritise specialised formulated foods (SFF) like lipid-based nutritional supplements (eg, ready-to-use supplemental foods or ready-to-use therapeutic food (RUTF)) over fortified-blended flours, especially in emergency contexts.^6^

The new guidelines also recommend prioritising SFFs for children with moderate wasting presenting with additional risk factors.^6 7^ Among the individual factors to be considered in the higher-priority category are children with MUAC 115–119 mm, weight-for-age (WAZ) z-score of <−3, age less than 24 months, a history of severe wasting, relapse from a previous wasting episode or significant comorbidities. Social risk factors to be considered include maternal death or poor maternal health and well-being.

However, implementing these multiple risk stratification simultaneously is operationally challenging in large-scale programmes. For example, in many settings where malnutrition treatment is delivered, a child’s history of clinical or social vulnerabilities is rarely available. And introducing additional criteria such as WAZ would require more reliable systematic age assessment and the use of lookup tables, which adds operational complexity to already overworked health staff. This is precisely why programmes have long relied on anthropometric indicators such as MUAC and WHZ that can be measured directly at health centres without requiring precise age assessment. For these reasons, it is essential to describe and understand the characteristics of children with moderate wasting enrolled by MUAC 115–124 mm, as they currently represent the population most feasibly reached by community-based programmes.

In recent years, a number of simplified protocols integrating treatment for severe and moderate wasting have emerged with the aim of detecting and treating children earlier in the wasting process. All children with severe or moderate wasting defined as MUAC <125 mm or nutritional oedema are admitted for treatment in a single programme and using a single nutritional supplement, RUTF, for treatment at a progressively reduced dosage according to the severity of malnutrition. OptiMA (Optimising Acute Malnutrition treatment) is one such approach that has demonstrated non-inferiority in a randomised control trial^3 8^ and has shown promising results in prospective observational cohorts.^9 10^ Other simplified, combined protocols have shown similarly promising results as well in multiple contexts.^11^,^16^

The OptiMA simplified protocol has been implemented at scale in both urban and rural contexts across West Africa, including prospective observational cohorts in Ngouri, Chad; Bamako, Mali and Mirriah, Niger. We conducted a secondary analysis of the database created from routinely collected programme data to describe the proportion of children admitted for treatment with moderate wasting by MUAC 115–124 mm who met one or more of the WHO-identified risk factors at admission (specifically MUAC 115–119 mm, WAZ <–3 and/or age less than 24 months). We also examined the association of these risk factors with treatment failure such as mortality, default and non-response and compared treatment outcomes of children in the higher-priority moderate wasting group to children in the lower-priority moderate wasting group across recovery, default, non-response, hospitalisation and death.

## Methods

### Study design

This study was a multicountry prospective observational cohort study among children aged 6–59 months with uncomplicated wasting by MUAC <125 mm or oedema treated weekly in outpatient health centres with the OptiMA protocol.

### Study settings

The OptiMA protocol was implemented at the district level in three locations: Ngouri (Chad), Bamako (Mali) and Mirriah (Niger).

#### Ngouri, Chad

The OptiMA protocol was set up in 34 health centres—32 of which were in Ngouri health district, Lake region, Chad and two health centres in neighbouring Isseirom health district. For simplicity’s sake, ‘Ngouri’ will refer to all 34 health centres participating in the study. The Lake Region of Chad is affected by a protracted, complex humanitarian crisis, caused by conflicts involving non-state armed groups, accelerated population growth, climate change and socioeconomic disparities. The total population of Ngouri in 2024 was estimated at 206 928, of whom 39 006 were children 6–59 months of age. In 2024, the prevalence of global acute malnutrition (GAM) among children in the Lake region was 11.9% (95% CI 9.0% to 15.6%) and 2.8% (95% CI 1.6% to 4.8%) for SAM.^17^ The integrated food insecurity classification system categorised the Lake region as ‘seriously food insecure’ in 2023.^18^

#### Bamako, Mali

Bamako, the capital of Mali, has a population of approximately 4 500 000, of whom 730 000 were children under 5 years of age.^19^ The under 5 mortality rate in Mali is 0.27 per 10 000 persons, with a similar rate of 0.26 per 10 000 in Bamako. The OptiMA protocol was implemented in the 12 health centres of district 1 and 8 health centres in district 2 in Bamako. In 2024, the prevalence of GAM among children in Bamako was 8.7% (95% CI 6.90% to 10.50%) and 1.3% (95% CI 0.7% to 2.3%) for SAM.^20^ In September 2024, Bamako experienced a sharp deterioration in security due to unprecedented attacks by armed groups.

#### Mirriah, Niger

In Mirriah, the OptiMA protocol was implemented in 21 health centres. The Mirriah Health District is one of the most populous in Niger, with an estimated population of 730 000 in 2023, of whom approximately 23.11% are children under the age of 5. The Zinder region, where Mirriah is located, is particularly affected by malnutrition. It recorded one of the highest observed prevalences of acute malnutrition with 10.5% (95% CI 9.3% to 11.9%) of GAM children and 3.2% (95% CI 2.6% to 3.9%) of SAM in 2022, as defined by MUAC <115 mm or the presence of oedema.^21^ Between 2022 and 2024, Niger, including the Zinder region, experienced a significant deterioration in food security, with over 4.4 million people, approximately 18% of the analysed population, facing Crisis level (CH phase 3) or worse acute food insecurity during the June–August 2022 period.^22^ This situation was driven by escalating conflict, flooding that damaged crops and livelihoods, and high food prices linked to the poor 2021–22 harvest and global economic shocks.

### Study period

In Ngouri, the study period spanned from 13 January 2022 to 31 December 2024, covering three consecutive years. In Bamako, data were collected from 1 January 2023 to 29 November 2024, covering almost 2 years of implementation. In Mirriah, data collection began in July 2023, and continued through 31 December 2024.

### Study population

This analysis focused on a subset of children aged 6–59 months enrolled in the OptiMA cohort and who had a MUAC between 115 and 124 mm at admission. Children who were admitted for treatment with severe acute malnutrition, defined in the OptiMA protocol as having a MUAC <115 mm or nutritional oedema, were excluded from this analysis. Importantly, each record represented a treatment episode rather than an individual child, as unique identifiers were not always reliable to distinguish new admissions from relapses across years.

Higher-priority episodes of moderate wasting were defined based on the 2023 WHO guidelines on the management of wasting with at least one of the following admission criteria: MUAC between 115–119 mm, WAZ <−3 Z-score or age <24 months.

Lower-priority episodes of moderate wasting were those with MUAC 120–124 mm aged between 24 and 59 months with WAZ >−3. The other criteria defined by the WHO guidelines such as history of severe wasting, having relapsed to moderate wasting, significant comorbidities or social factors were not available in the routinely collected data.

### OptiMA protocol

Children were considered eligible for enrolment if they were aged 6–59 months and presented spontaneously to any outpatient clinic in the study area with a MUAC <125 mm or mild or moderate bilateral pitting oedema without medical complications and passed an appetite test on the day of inclusion. Children with serious illness or 3+oedema are admitted with an OptiMA number and transferred to hospital.

Children were followed at weekly consultations at primary healthcare facilities, where they received a clinical exam that included anthropometric measurements, and given a weekly RUTF ration relative to the severity of their malnutrition. The OptiMA RUTF ration was calibrated to the child’s degree of wasting based on the combination of MUAC status and weight. Thus, more nutritional support was given to the most severely malnourished and gradually reduced as the child’s MUAC and weight increased. Specifically, moderately wasted children with MUAC 115–119 mm, either at admission or during the course of treatment, received 125–190 kcal/kg per day, and children with MUAC ≥120 mm received 50–166 kcal/kg per day (with a minimum of one sachet per day) until discharge from the programme.

All children underwent malaria rapid testing on inclusion and at any point during their follow-up if clinical signs of malaria were detected. All children with a positive malaria rapid diagnostic test were prescribed an artemisinin-combination treatment. Amoxicillin 90 mg/kg per day for 7 days was prescribed for all children with MUAC <120 mm or oedema at admission. Albendazole was given to children 9 months of age or older at the fourth follow-up visit if they had no deworming in the previous 4 months. Community health workers visited the homes of children who missed multiple consultations.

The criteria for nutritional recovery was MUAC ≥125 mm and no oedema for two consecutive weeks (with a minimum stay in the programme for 4 weeks). Children with a MUAC ≥125 mm and WHZ <−2 at inclusion were managed instead according to the current national protocol.

### Study outcomes

The primary outcome was the proportion of moderate wasting episodes (MUAC 115–124 mm) who met one or more WHO-defined criteria at admission (MUAC between 115 and 119 mm, WAZ < −3 Z-score, age <24 months).

Other outcomes were nutritional recovery, defined as achieving MUAC ≥125 mm without oedema on two consecutive visits within 12 weeks of admission and with a minimum stay in the programme for 4 weeks. Secondary outcomes included non-response (failure to reach recovery criteria within 12 weeks), default (missing ≥2 consecutive visits), death during treatment.

### Data collection procedures and monitoring

Data collection and monitoring procedures were standardised across all three study settings to ensure consistency and comparability of programmatic outcomes. Sociodemographic, clinical, and anthropometric data were collected by Ministry of Health staff supervised by a project manager using a modified version of the national programme individual outpatient record. A specific form was used for hospitalisation data. The staff involved in data collection were trained on the study protocol before participants’ enrolment. The child’s weight, MUAC, temperature, clinical symptoms and amount of RUTF ration were recorded at each weekly visit. Children’s length was measured at admission and once a month thereafter. Weight was measured to the nearest 100 g with a Salter scale, and length was measured to the nearest 0.5 cm on a height board with the child in a supine position (or standing if taller than 85 cm). MUAC was measured to the nearest mm with a MUAC bracelet demarcated in 1 mm increments. At each visit, supervisors ensured that scales were correctly calibrated and MUAC bracelets and height boards were in good condition.

All collected data were then anonymised before being entered into a database using CommCare. Data quality was ensured through routine verification processes: the project data manager generated weekly queries to identify missing, inconsistent or outlier values, which were then reviewed and corrected by nurse supervisors at the site level. In addition, annual international monitoring visits were conducted in each project site to assess data accuracy and protocol adherence.

### Data analysis

Descriptive statistics were used to summarise the characteristics of the study population at admission. The proportion of each individual risk criterion and at least one criterion was estimated by settings. Categorical variables were described as frequencies and percentages, and continuous variables as means (SD) or medians (IQRs), as appropriate. In cases of missing anthropometric data, we followed a conservative approach: children aged 6–23 months or with a MUAC between 115–119 mm with missing WAZ were systematically classified as higher-priority moderate wasting. However, children aged 24–59 months with a MUAC between 120–124 mm and missing WAZ data could not be definitively classified as either higher-priority or lower-priority and were therefore excluded from the analysis (n=18).

Higher-priority and lower-priority episodes of moderate wasting are described by setting in terms of programme outcomes, including recovery, non-response, defaulting and death. Anthropometric improvement such as weight and MUAC gain, hospitalisation were also compared between the higher-priority and lower-priority groups.

To identify independent predictors of adverse outcomes, a multinomial logistic regression model was fitted, using recovery as the reference outcome. This model provided adjusted ORs (aORs) and 95% CIs to estimate the likelihood of default, non-response or death relative to the odds of recovery for each risk criterion (WAZ <–3, MUAC category, age <24 months). Type of area (rural=Ngouri and Mirriah; urban=Bamako) was included in the multivariable model to control for potential confounding, as it is associated with both nutritional status (WAZ) and mortality risk. Sex was also tested as a potential confounder but was not retained in the final model as it did not significantly influence the associations. Statistical significance was set at p<0.05. Prior to final model selection, we tested for multicollinearity using variance inflation factors and examined potential interactions between risk factors; no significant collinearity or interactions were retained in the final model.

All analyses were conducted using R V.4.2.1.

## Results

A total of 87 961 episodes of moderate wasting defined by MUAC between 115 and 124 mm were included in this analysis, after excluding 560 cases due to duplication, missing data or medical contraindications (allergies). Most admissions occurred in Ngouri, Chad (55.5%), followed by Mirriah, Niger (33.1%) and Bamako, Mali (11.4%) (online supplemental file 1).

Table 1 shows the baseline characteristics of moderate wasting episodes treated with the OptiMA protocol in each setting. The median age of children at admission was 15 months (IQR: 10–22), with variations across settings: younger children were more represented in Bamako (median 12 months, IQR: 9–16) compared with Ngouri (16 months, IQR: 11–24) and Mirriah (15 months, IQR: 10–21). Overall, 54.6% of the cohort were girls, though proportions varied from 56.0% in Ngouri, 60.0% in Bamako and 50.5% in Mirriah. Anthropometric indicators also varied by setting. Overall, the mean WHZ was −2.3 (SD: 1.0), and 20.6% of children had WHZ <−3 at admission, with the highest proportion found in Bamako (26.8%) and the lowest in Mirriah (18.9%). Similarly, HAZ indicated high levels of stunting, particularly in Ngouri with 51.6% of HAZ<−3 compared with 11.9% in Bamako.

**Table 1:** Baseline characteristic of admitted for treatment with MUAC 115–124 mm under the OPtiMA protocol in Ngouri (Chad), Bamako (Mali) and Mirriah (Niger), 2022–2024 (N=87 961)

Characteristic	N	Overall, N=87 961	Ngouri (Chad), N=48 815	Bamako (Mali), N=10 062	Mirriah (Niger), N=29 084
Girl, n (%)	87 959	48 041 (54.6)	27 320 (56.0)	6034 (60.0)	14 687 (50.5)
Age in months, median (IQR)	87 961	15.0 (10.0, 22.0)	16.0 (11.0, 24.0)	12.0 (9.0, 16.0)	15.0 (10.0, 21.0)
Age category, n (%)	87 961				
6–11 months		27 720 (31.5)	13 821 (28.3)	4731 (47.0)	9168 (31.5)
12–23 months		40 476 (46.0)	21 419 (43.9)	4411 (43.8)	14 646 (50.4)
24–59 months		19 765 (22.5)	13 575 (27.8)	920 (9.1)	5270 (18.1)
Weight in kg, mean (SD)	87 789	7.0 (1.0)	7.0 (1.1)	6.9 (1.0)	6.9 (1.0)
Height, median (IQR)	87 947	70.5 (66.9, 75.0)	70.5 (66.5, 75.1)	71.0 (67.5, 75.0)	70.5 (67.0, 74.5)
Oedema, n (%)	87 947	0 (0.0)	0 (0.0)	0 (0.0)	0 (0.0)
MUAC in mm, mean (SD)	87 961	119.4 (2.7)	119.3 (2.7)	119.8 (2.5)	119.4 (2.7)
MUAC for age Z-score, mean (SD)	87 826	−2.6 (0.5)	−2.6 (0.5)	−2.3 (0.4)	−2.6 (0.5)
WHZ, mean (SD)	87 772	−2.3 (1.0)	−2.2 (1.1)	−2.4 (1.0)	−2.3 (0.9)
WHZ categories, n (%)	87 772				
<−3		18 075 (20.6)	9923 (20.4)	2671 (26.8)	5481 (18.9)
≥−3 and <−2		37 163 (42.3)	19 594 (40.2)	4201 (42.1)	13 368 (46.0)
HAZ, mean (SD)	87 812	−2.8 (1.6)	−3.0 (1.6)	−1.4 (1.4)	−2.9 (1.4)
HAZ categories, n (%)	87 812				
<−3		39 757 (45.3)	25 130 (51.6)	1186 (11.9)	13 441 (46.2)
≥−3 and <−2		21 724 (24.7)	2082 (20.8)	8205 (28.2)	11 437 (23.5)
WAZ, mean (SD)	87 789	−3.2 (1.0)	−3.3 (1.0)	−2.6 (0.9)	−3.2 (0.9)
WAZ categories, n (%)	87 789				
<−3		49 976 (56.9)	29 795 (61.1)	2908 (29.1)	17 273 (59.4)
≥−3 and <−2		27 795 (31.7)	14 160 (29.1)	4411 (44.2)	9224 (31.7)

HAZ, height-for-age Z; MUAC, mid-upper-arm circumference; OPtiMA, Optimising Acute Malnutrition Treatment; WAZ, weight-for-age Z; WHZ, weight for-height Z.

**Figure 1:**
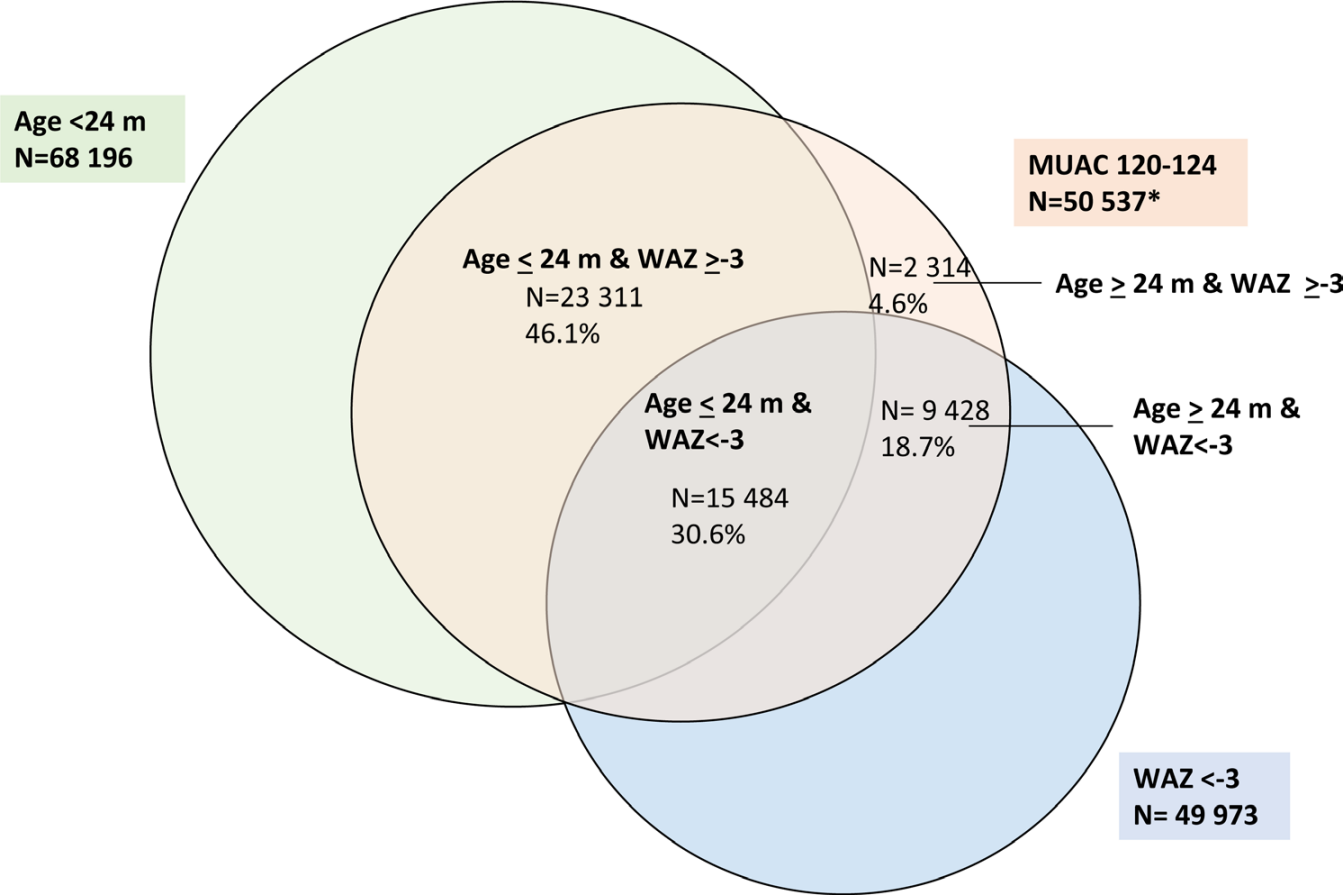
Venn diagram of MUAC, age and WAZ high-risk criteria among children with MUAC 120–124 mm in Ngouri (Chad), Bamako (Mali) and Mirriah (Niger), 2022–2024 (N=87 961). *18 children could not be classified as higher-priority or lower-priority Moderate Acute Malnutrition due to missing WAZ data (n=61); children aged 6–23 months with missing WAZ were classified as higher-priority Moderate Acute Malnutrition, following WHO guidance. MUAC, mid-upper-arm circumference; WAZ, weight-for-age Z.

The distribution of WHO higher-priority criteria among children with moderate wasting is summarised in table 2 and figure 1. Among children with moderate wasting defined by MUAC between 115 and 124 mm, 97.4% met at least one of the three higher-priority criteria defined by WHO analysed here (ie, age <24 month, WAZ <−3 or MUAC 115–119 mm), with slightly higher proportions in Mirriah (98.1%) and lower in Bamako (96.8%) (table 2). MUAC 115–119 mm was the most frequent single criterion, affecting 42.5% of all children. Among those with MUAC 120–124 mm, the Venn diagram illustrates the substantial overlap between age <24 months and WAZ <–3, 46.1% were also younger than 24 months, 18.7% had a WAZ <–3, and 30.6% simultaneously met both criteria. Only 4.6% presented with MUAC 120–124 mm without any additional risk factor. The distribution across sites varied; in Bamako, the combination of MUAC 120–124 mm and age <24 months predominated (71.6%), whereas WAZ <–3 was more common in Ngouri (Chad) and Mirriah (Niger). The Venn diagram also showed that 50.7% (25 572/50 537) of children with MUAC &20–124 mm had a WAZ >–3 and 23.2% (11 742/50 537) were aged more than 24 months.

**Table 2:** Distribution of WHO higher-priority criteria among moderately wasted children admitted for treatment with MUAC 115–124 mm under the OptiMA protocol in Ngouri (Chad), Bamako (Mali) and Mirriah (Niger), 2022–2024 (N=87 961)

Characteristic	N	Overall, N=87 961	Ngouri (Chad), N=48 815	Bamako (Mali), N=10 062	Mirriah (Niger), N=29 084
At least one WHO higher-priority criteria, n (%)	87 943*	85 629 (97.4)	9737 (96.8)	28 536 (98.1)	47 356 (97.0)
Distribution by criteria					
1 higher-priority criterion					
MUAC 115–119 mm at admission only, n (%)	87 961	37 406 (42.5)	21 919 (44.9)	3340 (33.2)	12 147 (41.8)
MUAC 120–124 mm and age <24 months only, n (%)	50 537*	23 311 (46.1)	10 801 (40.2)	4810 (71.6)	7700 (45.5)
MUAC 120–124 mm and WAZ <−3 only, n (%)	50 537*	9428 (18.7)	6626 (24.6)	362 (5.4)	2440 (14.4)
2 higher-priority criteria					
MUAC 120–124 mm and age <24 m and WAZ <−3, n (%)	50 537*	15 484 (30.6)	8010 (29.8)	1225 (18.2)	6249 (36.9)
Meeting none of the criterion					
MUAC 120–124 mm and no other higher-priority criteria, n (%)	50 537*	2314 (4.6)	1447 (5.4)	321 (4.8)	546 (3.2)

* 18 children could not be classified as higher-priority or lower-priority due to missing WAZ data (n=61); children aged 6–23 months with missing WAZ were classified as higher-priority, following WHO guidance.

MUAC, mid-upper-arm circumference; OPtiMA, Optimising Acute Malnutrition Treatment; WAZ, weight-for-age Z.

Table 3 presents a comparative analysis of children classified as higher-priority and lower-priority. Higher-priority and lower-priority children achieved comparably high recovery proportions across all sites, ranging from 85.0% (95% CI 84% to 86%) in Bamako to 90.3% (95% CI 90% to 91%) in Mirriah. Mortality (<0.4%), default (<7%) and non-response (2.5%) remained low in both groups. Median RUTF consumption was also similar between groups with an overall median of 45 sachets (IQR 37–60). Median length of stay was 28 days (IQR 28–42) in Ngouri and Bamako and 35 days (IQR 28–44) in Mirriah. Anthropometric improvement, measured by weight gain and MUAC gain, was slightly higher in higher-priority children overall. For example, mean weight gain in Ngouri was 4.2 g/kg/day (SD=2.1) in the higher-priority group versus 3.2 (SD=2.1) in the lower-priority group. Hospitalisation rates were low overall but consistently higher among higher-priority children across all sites. In Bamako, 6.3% of children in the higher-priority group were hospitalised at least once, compared with 3.4% in the lower-priority group.

**Table 3:** Programme outcomes, nutritional improvement and hospitalisation among children with lower-priority and higher-priority moderate wasting episodes admitted under the OptiMA protocol in Ngouri (Chad), Bamako (Mali) and Mirriah (Niger), 2022–2024 (N=87 943)*

Characteristic	N	Ngouri (Chad)		N	Bamako (Mali)		N	Mirriah (Niger)	
		Lower-priority, N=1447	Higher-priority, N=47 356		Lower-priority, N=321	Higher-priority, N=9737		Lower-priority, N=546	Higher-priority, N=28 536
Programme outcomes									
Discharge status, % (95% CI)	48 803			10 058			29 082		
Recovered		90.3 (89 to 92)	89.3 (89 to 90)		85.4 (81 to 89)	85.0(84 to 86)		88.3 (85 to 91)	90.3 (90 to 91)
Deceased		0.3 (0.1 to 0.8)	0.2 (0.2 to 0.3)		0.0(0.0 to 1.5)	0.1 (2.3 to 2.9)		0.2 (0.01 to 1.2)	0.4 (0.3 to 0.4)
Defaulters		1.5 (1.0 to 2.3)	1.9 (1.8 to 2.1)		1.9 (0.8 to 4.2)	2.6 (2.3 to 2.9)		6.8 (4.9 to 9.3)	3.5 (3.3 to 3.7)
Non responders		0.1 (0.02 to 0.6)	0.7 (0.6 to 0.7)		0.0 (0.0 to 1.5)	0.9 (0.7 to 1.1)		0.2 (0.01 to 1.2)	2.3 (2.1 to 2.4)
Others		7.7 (6.4 to 9.3)	7.8 (7.6 to 8.1)		12.8 (9.4 to 17.0)	11.5 (11.0 to 12.0)		4.6 (3.0 to 6.8)	3.6 (3.3 to 3.8)
MUAC >125 mm within 1 week, % (95% CI)	48 803	92.5 (91 to 94)	92.2 (92 to 92)	10 058	86.9 (83 to 90)	88.0 (87 to 89)	29 082	89.9 (87 to 92)	92.2 (92 to 92)
RUTF distributed in sachets, median (IQR)	48 744	45.0 (37.0, 49.0)	45.0 (37.0, 60.0)	9 999	45.0 (39.0, 48.0)	43.0 (36.0, 54.0)	29 079	45.0 (35.0, 49.0)	45.0 (39.0, 60.0)
MUAC 115–119 mm	21 876	–	60.0 (51.0, 72.0)	3 301	–	55.0 (46.0, 68.0)	12 146	–	57.0 (45.2, 75.0)
MUAC 120–124 mm	26 868	45.0 (37.0, 49.0)	39.0 (35.0, 44.0)	6 698	45.0 (39.0, 48.0)	39.0 (35.0, 45.0)	16 933	45.0 (35.0, 49.0)	42.0 (36.0, 49.0)
Length of stay in days, median (IQR)	48 706	28.0 (21.0, 28.0)	28.0 (28.0, 42.0)	9 988	28.0 (27.0, 33.0)	30.0 (28.0, 42.0)	29 057	28.0 (28.0, 35.0)	35.0 (28.0, 44.0)
Anthropometric gains									
Weight gain in g/kg per day, mean (SD)	48 083	3.2 (2.1)	4.2 (2.1)	9 755	2.9 (1.5)	4.1 (2.5)	28 800	3.5 (2.5)	3.9 (2.3)
MUAC gain in mm per day, mean (SD)	48 087	0.2 (0.1)	0.3 (0.1)	9 757	0.2 (0.1)	0.2 (0.1)	28 800	0.2 (0.2)	0.2 (0.1)
Hospitalisation									
Hospitalised at least once, % (95% CI)	48 803	0.8 (0.5 to 1.5)	2.0 (1.9 to 2.2)	10 058	3.4 (1.8 to 6.2)	6.3 (5.8 to 6.8)	29 082	7.0 (5.0 to 9.5)	4.3 (4.1 to 4.5)
Length of hospital stay in days, mean (SD)	977	5.2 (2.9)	6.3 (7.1)	622	6.2 (3.1)	5.8 (4.2)	1 258	6.6 (4.4)	4.9 (2.9)
In-hospital mortality, % (95% CI)	979	8.3 (0.4 to 40.0)	5.6 (4.3 to 7.3)	622	0.0 (0.0 to 32)	0.5 (0.1 to 1.6)	1 262	2.6 (0.1 to 15.0)	3.0 (2.2 to 4.2)

* 18 children could not be classified as higher-priority or lower-priority due to missing WAZ data (n=61); children aged 6–23 months with missing WAZ were classified as higher-priority, following WHO guidance.

MUAC, mid-upper-arm circumference; OPtiMA, Optimising Acute Malnutrition Treatment; RUTF, ready-to-use therapeutic food; WAZ, weight-for-age Z.

Table 4 shows a multinomial logistic regression model using recovery as the reference outcome. After adjustment, WAZ <–3 increased the odds of default (aOR=1.29; 95% CI 1.11 to 1.51) and non-response (aOR=1.29; 95% CI 1.02 to 1.62). A sensitivity analysis excluding the ‘type of area’ variable (online supplemental file 2) showed a significant association between WAZ <–3 and mortality, suggesting a confounding effect of geographic context. Children with low WAZ were disproportionately located in rural settings, likely attenuating the observed association in the adjusted model. Children with MUAC 120–124 mm had significantly lower odds of default (aOR=0.65; 95% CI 0.60 to 0.71) and non-response (aOR=0.26; 95% CI 0.23 to 0.30) compared with those with MUAC 115–119 mm. Age <24 months was independently associated with higher odds of non-response (aOR=2.59; 95% CI 2.11 to 3.17), but not with death or default. Urban residence was associated with lower mortality (aOR=0.26; 95% CI 0.11 to 0.59) but higher odds of defaulting (aOR=1.21; 95% CI 1.06 to 1.39).

**Table 4:** Multinomial logistic regression predicting exit status (recovery as reference) based on WHO higher-priority moderate wasting criteria (age, MUAC and WAZ categories) among children admitted under the OptiMA protocol in Ngouri (Chad), Bamako (Mali) and Mirriah (Niger), 2022–2024 (N=81 923)†*

Characteristic	n/N	%	Univariable multinomial regression			Multivariable multinomial regression		
			OR	95% CI	P value	ORa	95% CI	P value
Mortality								
Age categories								
24–59 months	55/18 288	0.3	—	—		—	—	
6–23 months	175/63 635	0.3	0.92	(0.68 to 1.25)	0.60	1.24	(0.89 to 1.72)	0.21
MUAC categories								
115–119 mm	106/34 689	0.3	—	—		—	—	
120–124 mm	124/47 234	0.3	0.83	(0.64 to 1.08)	0.17	0.98	(0.75 to 1.28)	0.88
WAZ categories*								
>−2	18/9209	0.2	—	—		—	—	
≥−3 and <−2	49/25 977	0.2	0.97	(0.56 to 1.66)	0.91	0.89	(0.52 to 1.54)	0.68
<−3	151/46 676	0.3	1.68	(1.03 to 2.74)	0.038	1.50	(0.91 to 2.48)	0.11
Type of area								
Rural	220/73 024	0.3	—	—		—	—	
Urban	10/8899	0.1	0.37	(0.20 to 0.70)	0.002	0.26	(0.11 to 0.59)	0.001
Defaulters								
Age categories								
24–59 months	535/18 288	3.0	—	—		—	—	
6–23 months	1687/63 635	2.7	0.91	(0.83 to 1.01)	0.066	0.97	(0.87 to 1.08)	0.58
MUAC categories								
115–119 mm	1182/34 689	3.4	—	—		—	—	
120–124 mm	1040/47 234	2.2	0.63	(0.58 to 0.68)	<0.0001	0.65	(0.60 to 0.71)	<0.0001
WAZ categories*								
>−2	201/9209	2.2	—	—		—	—	
≥−3 and <−2	596/25 977	2.3	1.05	(0.90 to 1.24)	0.52	1.04	(0.88 to 1.22)	0.65
<−3	1395/46 676	3.0	1.39	(1.20 to 1.61)	<0.0001	1.29	(1.11 to 1.51)	0.001
Type of area								
Rural	1967/73 024	2.7				—	—	
Urban	255/8899	2.9	1.06	(0.93 to 1.21)	0.39	1.21	(1.06 to 1.39)	0.006
Non-responders								
Age categories								
24–59 months	108/18 288	0.6	—	—		—	—	
6–23 months	937/63 635	1.5	2.51	(2.05 to 3.06)	<0.0001	2.59	(2.11 to 3.17)	<0.0001
MUAC categories								
115–119 mm	774/34 689	2.2	—	—		—	—	
120–124 mm	271/47 234	0.6	0.25	(0.22 to 0.29)	<0.0001	0.26	(0.23 to 0.30)	<0.0001
WAZ categories*								
>−2	86/9209	0.9	—	—		—	—	
≥−3 and <−2	306/25 977	1.2	1.27	(1.00 to 1.61)	0.055	1.14	(0.90 to 1.46)	0.28
<−3	651/46 676	1.4	1.52	(1.21 to 1.90)	0.0003	1.29	(1.02 to 1.62)	0.034
Type of area								
Rural	957/73 024	1.3	—	—		—	—	
Urban	88/8899	1.0	0.75	(0.60 to 0.94)	0.01	0.81	(0.65 to 1.01)	0.067

Note: Recovery is the reference category. aORs represent the risk of each adverse outcome (death, default, non-response) relative to the odds of recovery. An aOR >1 indicates a higher risk of that outcome compared with recovery.

* 61 missing WAZ data.

† Children included in the multinomial analysis were those classified as recovered, deceased, defaulters or non-responders, based on recalculated discharge status after checking discharge criteria. Children classified as ‘others’ (eg, erroneous discharges, early discharges or transfers) were excluded from this analysis.

aOR, adjusted OR; MUAC, mid-upper-arm circumference; OPtiMA, Optimising Acute Malnutrition Treatment; WAZ, weight-for-age Z.

## Discussion

This multicountry analysis of over 87 000 episodes of moderate wasting managed with the OptiMA protocol across three districts in West and Central African countries provides one of the first multicountry assessments of WHO’s 2023 risk-stratification criteria for moderate wasting in programmatic settings. The results show that nearly all children (97%) with MUAC between 115 and 124 mm at admission met at least one of the three operationally measurable WHO high-priority criteria (ie, MUAC 115–119 mm, age <24 months or WAZ <–3). Among children admitted with MUAC 120–124 mm, 46.1% were also aged <24 months, 18.7% had a WAZ <–3, and 30.6% met both risk criteria. Recovery proportions were high (85%–90%) across both higher-priority and lower-priority groups, mortality remained low (<0.4%) and hospitalisation proportions were under 7%. These results were consistent across rural and urban contexts. In multivariable analysis, WAZ <–3 was associated with increased risk of default (aOR=1.29; 95% CI 1.11 to 1.51) and non-response (aOR=1.29; 95% CI 1.02 to 1.62). While MUAC 120–124 mm was protective against these same outcomes, similarly high recovery and low mortality between the groups suggests that the intensity of treatment was appropriate for both. Age <24 months was only associated with non-response (aOR 2.59, 95% CI 2.11 to 3.17) but not with mortality or default.

The 2023 WHO guidelines recommend prioritising moderately wasted children with additional risk criteria for treatment with SFF.^6^ However, the feasibility of implementing these multiple criteria in large-scale malnutrition treatment programmes remains questionable. Recent WHO operational guidance also acknowledges the considerable challenges associated with applying multifactor risk stratification in routine health systems, especially in low-resource contexts, due to measurement complexity and increased staff workload.^7^ From an operational perspective, that nearly all children met one or more of the WHO-defined risk criteria highlights the limited utility of additional risk stratification in MUAC-based programmes. Moreover, programme outcomes were similar between higher-priority and lower-priority groups across all sites: recovery exceeded 85%, mortality remained below 0.4%, median length of stay (28–35 days) and RUTF consumption (around 45 sachets) were comparable. These findings are in line with a recently published analysis of moderately wasted children treated in the MUAC-based ComPAS programme in rural Mali which also found that nearly all children admitted by MUAC 115–124 mm (95%) had one or more risk criteria, and that there was no difference in terms of recovery between the higher-priority and lower-priority groups.^23^ This would indicate that the WHO risk stratification did not correspond to greater treatment needs or poorer outcomes. It also reinforces the programmatic efficiency of simplified approaches such as OptiMA and ComPAS, which have demonstrated that treating all children with MUAC <125 mm or nutritional oedema with a single protocol using RUTF doses adapted to the severity of malnutrition can achieve high recovery rates while maintaining operational feasibility and cost-effectiveness in resource-limited settings.^24 25^

WAZ <−3 emerged as the strongest and most consistent independent predictor of adverse outcomes. After adjusting for age and MUAC at admission, WAZ <−3 was significantly associated with higher odds of default and non-response. Mortality was a rare outcome in this cohort (<0.4%), which is consistent with previous studies in similar contexts.^23^ This low frequency represents a limitation in terms of statistical power for our multivariable model. While the association with death was marginally significant in the final model (aOR=1.50; 95% CI 0.91 to 2.48, p=0.11), a sensitivity analysis excluding the ‘type of area’ variable (urban/rural) made the association statistically significant, confirming WAZ <−3 as a strong predictive factor and type of context a confounding effect. These results corroborate evidence from pooled analyses and meta-analyses demonstrating that WAZ is a strong predictor of mortality and treatment failure.^26^,^30^ Specifically, in pooled analyses, children with WAZ <–3 had approximately a threefold increase in mortality risk compared with those with WAZ ≥–3 after adjusting for MUAC and WHZ.^26 27^ Because WAZ captures both wasting and stunting, it may identify children with more profound physiological vulnerability.^28^ However, practical barriers remain: computing WAZ requires both accurate age, which is not always easy to determine, and look-up tables, which make criteria harder to train and administer by lower-skilled health workers.^30^ It will be important, however, to determine whether children with WAZ <–3 but MUAC ≥125 mm, a group currently excluded from treatment, have as elevated risk of mortality as children with MUAC <125 mm.

While age <24 months was the most prevalent criterion, it was not a consistent predictor of poor outcomes, suggesting limited value for prioritisation by age once anthropometric criteria are considered. The finding of no association with death is in line with other published articles, and might be partly explained by the protective role of breastfeeding in this younger age group.^2 30 31^ While targeting children <24 months remains important, especially as nutritional deficits incurred during the first 2 years of life carry life-long consequences for health and development, excluding older children with low MUAC would have left many higher-priority children without treatment.^32^

The distinction between MUAC 115–119 mm and 120–124 mm did not independently predict mortality, though lower MUAC was associated with higher default and non-response. In MUAC-based and nutritional oedema protocols like OptiMA, early admission at higher MUAC thresholds might have likely mitigated this risk. Importantly, the finding that MUAC 120–124 mm was protective against default and non-response suggests that restricting admission to lower MUAC thresholds could exclude a substantial number of children still at elevated risk, thereby missing opportunities for early intervention and protection from deterioration. The risk factors associated with non-response identified in this analysis are also consistent with previously published findings, who also found that younger age, lower MUAC and lower WAZ at admission were significant predictors of failure to meet recovery criteria.^33 34^

Hospital referrals remained low across all sites (<7%), confirming that most children with moderate wasting can be effectively managed as outpatients under simplified MUAC-based protocols even with the high morbidity found in moderately wasted children.^35^ The generally low hospitalisation and mortality observed in this study reinforce the benefits of early detection and outpatient management of wasting, as previously reported in other West and Central African contexts.^10 36^ However, hospitalisation was significantly more frequent among higher-priority children in Chad and Mali but this trend was not observed in Niger, suggesting that hospitalisation remains highly dependent on site-specific differences and contexts.

This analysis benefits from a large sample size (>87 000 episodes of moderate wasting) and data drawn from three geographically and programmatically diverse contexts, strengthening both internal and external validity. Standardised data collection procedures and close supervision ensured consistency and data quality across sites. However, several limitations should be acknowledged. First, children with MUAC ≥125 mm but WHZ between –3 and –2 were not included, which reduces the representativeness of the broader population of children with moderate wasting. Repeating a similar analysis among children admitted with WHZ between −3 and −2 could be valuable. Second, we could not include other WHO-defined risk factors, such as relapse, chronic illness, disability or social vulnerability, due to limited routine data. This not only limits the representativeness of the WHO-defined higher-priority population but also potentially reduces the robustness of the multivariable model. It is known that clinical and socioeconomic factors such as fever, diarrhoea or lack of maternal education are also independent predictors of mortality and have not been studied in this analysis.^37^,^39^ Third, it was not possible to reliably distinguish new admissions from relapses across different years of data collection, as unique identifiers were inconsistently recorded. For this reason, the analysis was conducted at the level of moderate wasting episodes rather than individual children. Finally, we acknowledge that anthropometry was not double-blind measured, but this limitation is addressed by the very large sample size which smooths out any random error measurements.

This multicountry analysis demonstrates that nearly all of the children with moderate wasting admitted under a MUAC-based simplified protocol met at least one WHO risk criterion. While age <24 months was the most common risk factor, WAZ <–3 was the strongest independent predictor of adverse outcomes. The positive treatment outcomes observed across higher-priority and lower-priority groups support the continued expansion of simplified, MUAC-based protocols as an effective and pragmatic strategy to operationalise WHO recommendations. Future research should study the mortality risk profile of children with WAZ <–3 but MUAC≥125 mm, a group currently excluded from treatment, as well as explore practical ways to include children with WAZ <–3 in treatment.

## Supplementary material

10.1136/bmjgh-2025-023264 online supplemental file 1

10.1136/bmjgh-2025-023264 online supplemental file 2

10.1136/bmjgh-2025-023264 online supplemental file 3

## Data Availability

Data are available on reasonable request.
