# Supplementary material for: Assessing WHO prioritisation criteria for children 6–59 months treated for moderate wasting in a MUAC-based protocol: a multicountry analysis in West and Central Africa
Source: BMJ Glob Health. 2026 Jul 10;11(7):e023264. doi: 10.1136/bmjgh-2025-023264 (PMC13358235; doi:10.1136/bmjgh-2025-023264)
Supplement: online supplemental file 1 [file bmjgh-11-7-s001.docx]

**Number of MAM episodes admitted with MUAC between 115-124 mm**

**N = 88 521**

560 excluded episodes:

- 372 duplicates

- 2 missing age

- 33 missing consent

- 153 with milk or peanut allergies

**Number of MAM episodes included for analysis N = 87 961 (99.4%)**

**Ngouri (Chad)**

**Episodes : 48 815 (55.5%)**

Including in

- 2022*: 15 414 episodes

- 2023: 16 512 episodes

- 2024: 16 889 episodes

**Bamako (Mali)**

**Episodes : 10 062 (11.4%)**

Including in

- 2023: 4 327 episodes

- 2024^§^: 5 735 episodes

**Mirriah (Niger)**

**Episodes : 29 084 (33.1%)**

Including in

- 2023^$^: 8 430 episodes

- 2024: 20 654 episodes

**Supplementary File 1: Flowchart of children admitted with a MUAC between 115 and 124 mm included and excluded from analysis.** *Ngouri: Year 2022 started from January 13^th^ to December 31 ^th^; ^§^Bamako: Year 2024 started from January 1^st^ to November 29^th^ ; ^$^ Mirriah: Year 2023 started From July 5 ^th^ to December 31^th^.
