# Supplementary material for: Assessing WHO prioritisation criteria for children 6–59 months treated for moderate wasting in a MUAC-based protocol: a multicountry analysis in West and Central Africa
Source: BMJ Glob Health. 2026 Jul 10;11(7):e023264. doi: 10.1136/bmjgh-2025-023264 (PMC13358235; doi:10.1136/bmjgh-2025-023264)
Supplement: online supplemental file 2 [file bmjgh-11-7-s002.docx]

**Supplementary File 2 : Multinomial logistic regression predicting exit status (recovery as reference) based on WHO high risk MAM criteria (age, MUAC category and WAZ) among children admitted under the OptiMA protocol in** **Ngouri (Chad), Bamako (Mali) and Mirriah (Niger), 2022-2024 (n=81,923*).**

|  |  |  | **Univariable multinomial regression** | | | **Multivariable Multinomial regression** | | |
| --- | --- | --- | --- | --- | --- | --- | --- | --- |
| **Characteristic** | **n/N** | **%** | **OR** | **95% CI** | **p-value** | **ORa** | **95% CI** | **p-value** |
| ***Deceased*** |  |  |  |  |  |  |  |  |
| Age categories |  |  |  |  |  |  |  |  |
| 24-59 months | 55/18,288 | 0·3 | — | — |  | — | — |  |
| 6-23 months | 175/63,635 | 0·3 | 0·92 | [0·68 - 1·25] | 0·60 | 1·20 | [0·86-1·67] | 0·28 |
| MUAC categories |  |  |  |  |  |  |  |  |
| 115-119 mm | 106/34,689 | 0·3 | — | — |  | — | — |  |
| 120-124mm | 124/47,234 | 0·3 | 0·83 | [0·64-1·08] | 0·17 | 0·96 | [0·74-1·27] | 0·80 |
| WAZ categories* |  |  |  |  |  |  |  |  |
| >-2 | 18/9,209 | 0·2 | — | — |  | — | — |  |
| <-3 | 151/46,676 | 0·3 | 1·68 | [1·03- 2·74] | 0·038 | 1·75 | [1·06-2·89] | 0·029 |
| >-3/<-2 | 49/25,977 | 0·2 | 0·97 | [0·56-1·66] | 0·91 | 0·97 | [0·57-1·67] | 0·93 |
| ***Defaulters*** |  |  |  |  |  |  |  |  |
| Age categories |  |  |  |  |  |  |  |  |
| 24-59 months | 535/18,288 | 3·0 | — | — |  | — | — |  |
| 6-23 months | 1,687/63,635 | 2·7 | 0·91 | [0·83-1·01] | 0·066 | 0·98 | [0·88-1·09] | 0·70 |
| MUAC categories |  |  |  |  |  |  |  |  |
| 115-119 mm | 1,182/34,689 | 3·4 | — | — |  | — | — |  |
| 120-124mm | 1,040/47,234 | 2·2 | 0·63 | [0·58-0·68] | <0·0001 | 0·65 | [0·60-0·71] | <0·0001 |
| WAZ categories* |  |  |  |  |  |  |  |  |
| >-2 | 201/9,209 | 2·2 | — | — |  | — | — |  |
| <-3 | 1,395/46,676 | 3·0 | 1·39 | [1·20-1·61] | <0·0001 | 1·25 | [1·07-1·46] | 0·005 |
| >-3/<-2 | 596/25,977 | 2·3 | 1·05 | [0·90-1·24] | 0·52 | 1·02 | [0·87-1·20] | 0·82 |
| ***Non-responders*** | |  |  |  |  |  |  |  |
| Age categories |  |  |  |  |  |  |  |  |
| 24-59 months | 108/18,288 | 0·6 | — | — |  | — | — |  |
| 6-23 months | 937/63,635 | 1·5 | 2·51 | [2·05-3·06] | <0·0001 | 2·57 | [2·09-3·15] | <0·0001 |
| MUAC categories |  |  |  |  |  |  |  |  |
| 115-119 mm | 774/34,689 | 2·2 | — | — |  | — | — |  |
| 120-124mm | 271/47,234 | 0·6 | 0·25 | [0·22-0·29] | <0·0001 | 0·26 | [0·23-0·30] | <0·0001 |
| WAZ categories* |  |  |  |  |  |  |  |  |
| >-2 | 86/9,209 | 0·9 | — | — |  | — | — |  |
| <-3 | 651/46,676 | 1·4 | 1·52 | [1·21-1·90] | 0·0003 | 1·33 | [1·06-1·67] | 0·016 |
| >-3/<-2 | 306/25,977 | 1·2 | 1·27 | [1·00-1·61] | 0·055 | 1·16 | [0·91-1·48] | 0·22 |

HAZ=height-for-age Z. MUAC=mid-upper-arm circumference. WAZ=weight-for-age Z. WHZ=weight for-height Z.

^$^ Children included in the multinomial analysis were those classified as recovered, deceased, defaulters, or non-responders, based on recalculated discharge status after checking discharge criteria. Children classified as “others” (e.g., erroneous discharges, early discharges, or transfers) were excluded from this analysis. *61 missing WAZ data
