# Supplementary material for: Assessing WHO prioritisation criteria for children 6–59 months treated for moderate wasting in a MUAC-based protocol: a multicountry analysis in West and Central Africa
Source: BMJ Glob Health. 2026 Jul 10;11(7):e023264. doi: 10.1136/bmjgh-2025-023264 (PMC13358235; doi:10.1136/bmjgh-2025-023264)
Supplement: online supplemental file 3 [file bmjgh-11-7-s003.docx]

### BMJ Global Health Author Reflexivity Statement

Adapted from Morton, B., Vercueil, A., Masekela, R., Heinz, E., Reimer, L., Saleh, S., Kalinga, C., Seekles, M., Biccard, B., Chakaya, J., Abimbola, S., Obasi, A. and Oriyo, N. (2022), Consensus statement on measures to promote equitable authorship in the publication of research from international partnerships. Anaesthesia, 77: 264-276. <https://doi.org/10.1111/anae.15597>

| **Study conceptualisation** | |
| --- | --- |
| 1. How does this study address local research and policy priorities? | This study responds to key priorities identified by Ministries of Health and national nutrition clusters in Chad, Niger, and Mali, where moderate wasting remains highly prevalent and existing programmes struggle with resource constraints. Understanding how WHO high-risk criteria apply in MUAC-based simplified programmes directly informs national decisions on adopting or scaling simplified or combined protocols for acute malnutrition management. |
| 1. How were local researchers involved in study design? | This work is a secondary analysis of data generated through the OptiMA implementation projects led by ALIMA and national health authorities in Chad, Niger, and Mali. As such, local clinicians, programme managers and research coordinators were directly involved in the design and operational implementation of the OptiMA protocol and its data collection procedures. However, because the present study is a secondary analysis conducted retrospectively using routine programme data, there was no additional study design phase requiring further involvement of local researchers. |
| **Research management** | |
| 1. How has funding been used to support the local research team(s)? | Funding covered salaries of field data managers, nurse supervisors, and monitoring officers who are part of local teams. It also supported national training workshops, supervision visits, and local research coordination in each country. |
| **Data acquisition and analysis** | |
| 1. How are research staff who conducted data collection acknowledged? | All frontline nurses, nutrition assistants, and supervisors involved in data collection are acknowledged in the manuscript. Their contribution is also formally recognized through inclusion of project coordinators as co-authors. |
| 1. How have members of the research partnership been provided with access to study data? | Local research teams in all three countries have full access to the curated dataset via secure shared platforms managed by ALIMA. |
| 1. How were data used to develop analytical skills within the partnership? | The project included training sessions for local data managers and clinical research associate on data cleaning, using CommCare exports etc.  This work is a secondary analysis of programme data routinely collected by ALIMA and Ministries of Health teams in Chad, Niger, and Mali. The secondary analysis was conducted at the University of Bordeaux (France) and no additional project-specific funding was provided for local research staff for the purposes of this analysis. |
| **Data interpretation** | |
| 1. How have research partners collaborated in interpreting study data? | Findings were reviewed jointly during online meetings. National teams led the contextual interpretation of site-specific results, particularly patterns in age distribution, stunting, and hospitalization, ensuring that conclusions reflected local realities. |
| **Drafting and revising for intellectual content** | |
| 1. How were research partners supported to develop writing skills? | Country teams contributed directly to sections on methods, limitations, and contextual interpretation. |
| 1. How will research products be shared to address local needs? | Results will be disseminated through national nutrition cluster meetings, Ministry of Health technical groups, and operational partners (UNICEF, WFP, NGOs). Simplified briefs will be prepared in French for use by district health teams. |
| **Authorship** | |
| 1. How is the leadership, contribution and ownership of this work by LMIC researchers recognised within the authorship? | LMIC researchers from Chad, Niger, and Mali are included as co-authors based on substantial contributions to study implementation, data management, interpretation. |
| 1. How have early career researchers across the partnership been included within the authorship team? | Early career national nutrition officers, data managers, and junior epidemiologists have been included as authors according to ICMJE criteria |
| 1. How has gender balance been addressed within the authorship? | The authorship team reflects gender diversity across both national and international contributors, with several women in lead analytical, coordination, and writing roles. |
| **Training** | |
| 1. How has the project contributed to training of LMIC researchers? | The project delivered training on godd clinical practices (GCP), anthropometric measurement, clinical assessment, digital data collection using CommCare and data quality. |
| **Infrastructure** | |
| 1. How has the project contributed to improvements in local infrastructure? | This project is implemented within the framework of ALIMA’s humanitarian programmes in Chad, Niger, and Mali. ALIMA supports programs in paediatric health and Nutrition since many years. Through these programmes, ALIMA has contributed to strengthening local health system infrastructure over many years. |
| **Governance** | |
| 1. What safeguarding procedures were used to protect local study participants and researchers? | All activities adhered to national ethical guidelines and GCP. Personal identifiers were not collected; all data were anonymised. Field staff were trained in child safeguarding, informed consent procedures, adverse event reporting, and secure data handling. Supervision protocols ensured safe working conditions for staff across all sites. |
